# Plasma C4d as marker for lupus nephritis in systemic lupus erythematosus

**DOI:** 10.1186/s13075-017-1470-2

**Published:** 2017-12-06

**Authors:** Myriam Martin, Karolina I. Smoląg, Albin Björk, Birgitta Gullstrand, Marcin Okrój, Jonatan Leffler, Andreas Jönsen, Anders A. Bengtsson, Anna M. Blom

**Affiliations:** 1 Department of Translational Medicine, Section of Medical Protein Chemistry, Lund University, Inga Marie Nilsson’s Street 53, 205 02 Malmö, Sweden; 2 Rheumatology, Department of Clinical Sciences Lund, Lund University, Lund, Sweden; 3 Department of Medical Biotechnology, Intercollegiate Faculty of Biotechnology, University of Gdańsk and Medical University of Gdańsk, Gdańsk, Poland; 4 Telethon Kids Institute, University of Western Australia, Perth, Australia

**Keywords:** Systemic lupus erythematosus, Complement, C4d, Flare, Lupus nephritis

## Abstract

**Background:**

In the present study, we sought to evaluate the complement activation product C4d as a marker for lupus nephritis in systemic lupus erythematosus (SLE).

**Methods:**

C4d levels were determined by enzyme-linked immunosorbent assay in plasma samples of patients with established SLE using a novel approach based on detection of a short linear cleavage neoepitope. Cross-sectional associations were studied in 98 patients with SLE with samples taken at lower or higher respective disease activity. Temporal associations were investigated in 69 patients with SLE who were followed longitudinally for up to 5 years. Plasma samples from 77 healthy donors were included as controls.

**Results:**

C4d levels were negligible in healthy control subjects and significantly increased in patients with SLE in the cross-sectional study (*p* < 0.0001). C4d levels discriminated between higher and lower disease activity according to ROC curve analysis (*p* < 0.001), exhibiting a positive predictive value of 68%. At higher disease activity, C4d levels correlated with the modified Systemic Lupus Erythematosus Disease Activity Index (*p* = 0.011) and predominantly with lupus nephritis (*p* = 0.003), exhibiting a sensitivity of 79% to identify patients with nephritis. High C4d levels together with the presence of anti-dsDNA autoantibodies preceded and thus predicted future lupus nephritis in the longitudinal study (OR 5.4, 95% CI 1.4–21.3). When we considered only patients with renal involvement (19 of 69) during the longitudinal study, we found that high C4d levels alone could forecast recurrence of future lupus nephritis (OR 3.3, 95% CI 1.2–9.6).

**Conclusions:**

C4d appears to be a valuable marker for use in monitoring of patients with SLE, particularly for lupus nephritis. Importantly, C4d levels can predict impending flares of lupus nephritis and may thus be useful for informing treatment.

## Background

Systemic lupus erythematosus (SLE) is a chronic autoimmune disorder with complex etiology and multiorgan involvement causing clinical manifestations such as malar rash, arthritis, and renal disorders [1]. It is characterized by periods of illness and flares, which result in reduced quality of life and increased mortality as well as periods of low disease activity. Lupus nephritis (LN) is one of the most severe manifestations of SLE. There is no curative treatment for SLE, and current treatment strategies are aimed at minimizing organ damage.

Activation of the complement system is a hallmark of SLE. Low serum levels of complement components C3 and C4 have been used for over 50 years to indicate lupus activity and are included in the Systemic Lupus Erythematosus Disease Activity Index (SLEDAI). Because levels of C3 or C4 depend not only on complement activation but also on the rate of synthesis, the measurement of complement activation products has been suggested as a more specific SLE biomarker [2–4]. A variety of other biomarkers are used to diagnose SLE, to monitor disease activity, and to identify and/or predict specific organ involvement [5]. However, no biomarker covers all aspects of the different phenotypes of the disease, and some assays are prone to producing false-positive data owing to mistreatment of the samples.

In the present study, by applying our novel, robust, and feasible C4d enzyme-linked immunosorbent assay (ELISA), we show that C4d, which is the final cleavage product of C4 arising during complement activation, is a valuable marker to discriminate higher disease activity and especially LN. C4d levels correlate with SLEDAI and can forecast impending renal flares in patients with previous renal involvement.

## Methods

### Study participants

#### Patients with SLE

All patients were recruited from the Department of Rheumatology, Lund University, Lund, Sweden, and fulfilled at least four of the American College of Rheumatology classification criteria for SLE [6], which are listed together with demographics and treatments in Table 1. The cross-sectional study group included 98 patients with SLE whose disease activity was assessed prospectively using the Systemic Lupus Erythematosus Disease Activity Index 2000 (SLEDAI-2K) [7]. Two plasma samples were selected for each patient and collected at time points with higher and lower relative clinical disease activity. The longitudinal study group encompassed 69 patients followed periodically with a median (range) of 13 (2–42) visits. A time interval of 70±30 days was chosen to assess temporal associations. Fifty-four patients overlapped in both study groups. However, the sampling time points did not overlap. Disease activity was recorded at every visit using the SLEDAI-2K. Samples were defined as nephritis-positive when SLEDAI scores were given for any of the following: urinary cast, hematuria, proteinuria, or pyuria. Blood samples were centrifuged 1 h after sampling, and plasma was aliquoted and stored at −80 °C no longer than 2 h after venipuncture. Although we do not have genetic data for a majority of the patients, they were all screened for complement deficiency using functional tests. These showed that none of the patients had a complete deficiency in C3 or C4. However, we cannot rule out partial deficiencies.

**Table 1:** Demographics and clinical characteristics of the cross-sectional and longitudinal study groups

Characteristics	Study 1 (*n* = 98) Cross-sectional Higher/lower disease activity	Study 2 (*n* = 69) Longitudinal
Age at sampling, years, median (range)	41 (14–75)/43 (19–81)	39.2 (18–76)
Female sex, *n* (%)	88 (91)	63 (91.3)
Disease duration, years, median (range)	4 (0–40)/7 (0–43)	7.5 (0–41)
Follow-up duration, days, median (range)	N/A	778 (139–1792)
SLEDAI-2K	9 (2–28)/2 (0–12)	2 (0–24)
ACR classification criteria, *n*		
Malar rash	65	46
Discoid rash	29	19
Photosensitivity	61	43
Oral ulcer	29	24
Arthritis	82	60
Serositis	56	39
Lupus nephritis	45	34
Neurological disorder	8	3
Hematological disorder	53	38
Immunological disorder	74	53
Antinuclear antibodies	96	69
Treatment, *n*		
Antimalarial	36/35	46
Corticosteroid	64/64	55
Immunomodulatory	42/51	41

*ACR* American College of Rheumatology, *SLEDAI-2K* Systemic Lupus Erythematosus Disease Activity Index 2000

#### Healthy control subjects

Seventy-seven healthy control subjects were recruited in 2011 at the Department of Translational Medicine, Lund University, Malmö, Sweden. Fifty-two (68%) patients were female, and their median age was 38 years (range 21–74). Plasma samples were prepared according to the same protocol used for the patients’ samples.

### ELISA detecting soluble C4d

Nunc MaxiSorp 96-well plates (Invitrogen, Carlsbad, CA, USA) were coated with rabbit anti-human C4d neoepitope-specific antibody [8]. After quenching with washing buffer (50 mM Tris-HCl, 0.15 M NaCl, 0.1% Tween, pH 7.5) supplemented with 3% fish gelatin (Norland Products, Cranbury, NJ, USA), patient or control plasma and pooled plasma from healthy volunteers (lacking C4d) supplemented with *Escherichia coli*-expressed C4d standard in serial dilutions were diluted to 4% in PBS with 0.02% Tween-20 and 0.02 M disodium ethylenediaminetetraacetate dihydrate and added to the plate. Detection was achieved using mouse anti-human C4d antibody (catalogue number 253; Quidel, San Diego, CA, USA) followed by horseradish peroxidase-conjugated goat anti-mouse secondary antibody (Dako, Carpinteria, CA, USA). Plates were developed using *o*-phenylenediamine dihydrochloride as a substrate, and absorbance was measured at 490 nm using a Varian Cary 50 microplate reader (Agilent Technologies, Santa Clara, CA, USA). The lowest detection limit was 5.6 μg/L. Values below the detection limit were set to 0.001 mg/L for statistical calculations. The inter-assay coefficient of variation was 16.7%, and the intra-assay coefficient of variation was 13.2%.

### Standard laboratory tests and reference values

All routine laboratory tests necessary to assess disease activity were executed at the Unit of Clinical Immunology (Lund, Sweden). Until 2009, levels of C3 and C4 were measured with the ABX Pentra 400 assay (Horiba Medical, Irvine, CA, USA) using antihuman C3c (catalogue number Q0368; Dako) and antihuman C4 (catalogue number Q0369; Dako) antibodies. Thereafter, the IMMAGE 800 system (Beckman Coulter, Brea, CA, USA) was applied for nephelometry using antihuman C3 (catalogue number 446450; Beckman Coulter) and antihuman C4 (catalogue number 446490; Beckman Coulter) antibodies. A modified version of the SLEDAI 2000 (mSLEDAI), excluding scores for low levels of complement factors and/or anti-double-stranded DNA (dsDNA) antibodies, was also calculated. To create categorized variables, the following clinical routine reference values were applied: low C3 < 0.77 g/L and low C4 < 0.12 g/L.

### Statistical analyses

Statistical significance for nonparametric continuous data was calculated using the Mann-Whitney *U* test for two groups and the Kruskal-Wallis rank-sum test for more than two groups. Data are presented as medians with 25–75% quantiles plus whiskers. Correlations of nonparametric data were analyzed using Spearman’s rank-order correlation test. For some analyses, C4d levels were categorized according to ROC curve analyses (cross-sectional study 0.39 mg/L for higher disease activity and 0.42 mg/L for LN; longitudinal study 1.1 mg/L for LN). ROC curves and AUCs, together with positive predictive values (PPVs) and negative predictive values (NPVs), were determined to evaluate accuracy. McNemar’s test was used for comparison of two markers in terms of accuracy. ORs and 95% CIs were calculated to estimate the relative risk. A generalized estimating equation was used when estimating ORs or regression coefficients in models with correlated outcomes, which can arise when the same patient is included more than once. A statistical significance level of (*p* value) < 0.05 was defined as statistically significant. Analyses were carried out using JMP Pro 12 (SAS Institute, Cary, NC, USA) and IBM SPSS Statistics 23 (IBM, Armonk, NY, USA) software.

## Results

### C4d levels were increased in patients with SLE and associated strongly with higher disease activity in cross-sectional study

**Fig. 1:**
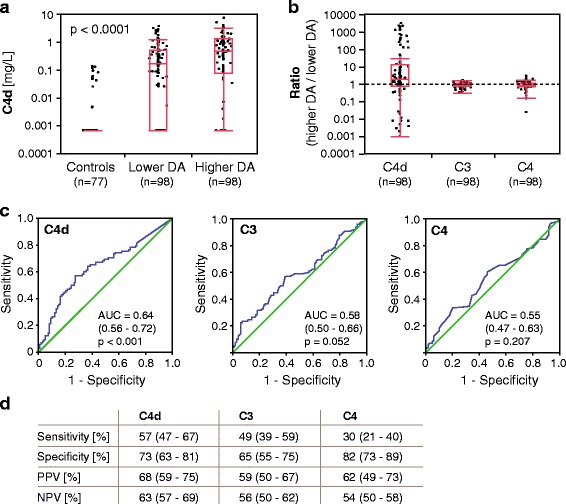
Plasma C4d levels are increased in patients with systemic lupus erythematosus (SLE) in the cross-sectional study, and C4d is convincing as an accurate marker for identifying higher disease activity (DA). **a** C4d levels in healthy control subjects (*n* = 77) and patients with SLE at lower (*n* = 98) and higher (*n* = 98) DA. Data are presented as medians with 25–75% quantiles plus whiskers, and significance was calculated using the Kruskal-Wallis rank-sum test. **b** Ratio of higher DA to lower DA of C4d, C3, and C4 levels in patients with SLE (*n* = 98). The *dashed line* indicates equal levels at higher and lower DA. Data are presented as medians with 25–75% quantiles plus whiskers. **c** Area under the ROC curve analysis shows that only C4d, but not C3 and C4, exhibits accuracy as a marker for higher DA. **d** Sensitivity, specificity, positive predictive value (PPV), and negative predictive value (NPV) for C4d, C3, and C4 as markers for higher DA. Data are presented with 95% CIs

C4d levels were measured with our in-house C4d ELISA [8]. In 63 of the 77 healthy control samples, C4d levels were below the detection limit. The level in the remaining 14 samples ranged from 0.005 mg/L to 0.138 mg/L (Fig. 1a). In patients with SLE, C4d levels were significantly increased, both in lower (0.17 mg/L, 0.001–0.51 mg/L) and in higher (0.49 mg/L, 0.08–1.31 mg/L) disease activity (Fig. 1a). Paired analysis of each patient revealed that C4d levels, on average, significantly increased threefold between lower and higher disease activity (*p* < 0.0001), whereas C3 levels decreased only 1.1-fold (lower activity 0.88 g/L, 0.72–1.05 g/L; higher activity 0.79 g/L, 0.64–1.01 g/L; *p* = 0.028) and C4 levels even insignificantly (lower activity 0.17 g/L, 0.13–0.21 g/L; higher activity 0.16 g/L, 0.11–0.21 g/L; *p* = 0.108). Thus, the magnitude of change was higher for C4d than for C3 and C4. Sixty-two patients exhibited higher C4d levels at higher disease activity, 7 patients exhibited unaltered levels, and 29 patients exhibited lower levels (Fig. 1b). C3 levels were lower in 59 patients at higher disease activity, unaltered in 1 patient, and higher in 38 patients. C4 levels were lower in 55 patients at higher disease activity, unchanged in 7 patients, and higher in 36 patients. As expected, at higher disease activity, C4d levels correlated negatively with C3 (*r*_s_ = −0.414, *p* < 0.0001) and C4 levels (*r*_s_ = −0.29, *p* < 0.0038).

Medications differed between patients (Table 1), but no significant correlation with C4d levels and different therapies was observed.

### C4d was an accurate marker to discriminate between higher and lower disease activity in cross-sectional study

Area under the ROC curve analysis confirmed that C4d displays statistically significant accuracy as a marker to differentiate higher from lower disease activity, whereas C3 and C4 do not (Fig. 1c). C4d levels were categorized according to ROC curve, and C3 and C4 levels were categorized according to routine clinical reference values. C4d had the highest sensitivity (57%), PPV (68%), and NPV (63%) as a marker for higher disease activity (Fig. 1d), whereas C4 exhibited the highest specificity (82%). High C4d levels have statistically superior accuracy as a marker of higher disease activity compared with low C4 levels (*p* < 0.0001 by McNemar’s test) and accuracy similar to low C3 levels (*p* = 0.345).

### C4d levels correlate with disease activity

**Fig. 2:**
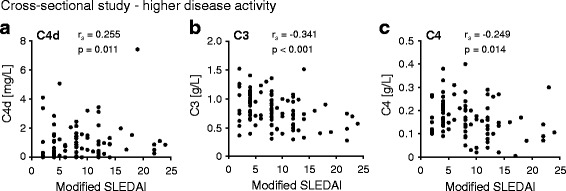
C4d, C3, and C4 levels correlate with modified Systemic Lupus Erythematosus Disease Activity Index (SLEDAI). Correlation of C4d (**a**), C3 (**b**), and C4 (**c**) levels with modified SLEDAI (excluding scores for low levels of complement factors and/or anti-double-stranded DNA antibodies) in the cross-sectional study at higher disease activity. Significance and correlation coefficient (*r*_s_) were calculated using Spearman’s rank-order correlation test

As anticipated, C4d levels were significantly associated with low complement and occurrence of anti-dsDNA antibodies (Table 2). Thus, the mSLEDAI, excluding scores for low levels of complement factors and anti-dsDNA antibodies, was applied. In the cross-sectional cohort, elevated levels of C4d were detected at higher disease activity and correlated significantly with mSLEDAI (Fig. 2a). As expected, levels of C3 and C4 inversely correlated with mSLEDAI. At lower disease activity, neither C4d nor C3 nor C4 levels correlated significantly with mSLEDAI. In the longitudinal study group, only C4d, but neither C3 nor C4 levels, correlated significantly with mSLEDAI (β = 0.05, *p* = 0.022).

**Table 2:** Systemic Lupus Erythematosus Disease Activity Index-qualifying symptoms and C4d levels at higher disease activity in the cross-sectional study (*n* = 98)

	Higher disease activity					Lower disease activity
	Feature present		Feature absent			
Clinical features	No. of subjects	C4d (mg/L)	No. of subjects	C4d (mg/L)	*p* Value	No. of subjects
Seizure	2	0.56 (0–1.12)	96	0.49 (0.09–1.32)	–	0
Psychosis	0	–	98	0.49 (0.08–1.31)	–	0
Organic brain syndrome	0	–	98	0.49 (0.08–1.31)	–	0
Visual disturbance	7	0.53 (0.35–0.88)	91	0.45 (0.05–1.32)	0.658	0
Cranial nerve disorder	0	–	98	0.49 (0.08–1.31)	–	0
Lupus headache	2	0.39 (0–0.77)	96	0.49 (0.09–1.32)	–	0
Cerebrovascular accident	2	0	96	0.51 (0.10–1.32)	–	0
Vasculitis	12	0.73 (0.33–1.44)	86	0.46 (0.05–1.16)	0.342	0
Arthritis	29	0.15 (0.001–1.03)	69	0.65 (0.21–1.33)	**0.033**	1
Myositis	1	0.19 (0.19–0.19)	97	0.51 (0.07–1.31)	–	0
Kidney involvement (urinary cast, hematuria, proteinuria, or pyuria)	34	0.89 (0.44–1.56)	64	0.34 (0 –1.12)	**0.003**	1
Rash	30	0.45 (0.10–1.15)	68	0.52 (0.05 –1.38)	0.745	4
Alopecia	7	0.33 (0.03–0.76)	91	0.51 (0.09 –1.32)	0.365	1
Oral ulcers	7	0.40 (0.11–1.24)	91	0.51 (0.05 –1.32)	0.868	0
Pleurisy	13	0.88 (0.34–2.16)	85	0.44 (0.05 –1.19)	0.172	0
Pericarditis	7	1.07 (0.12–2.85)	91	0.45 (0.05 –1.24)	0.237	0
Low complement (C3 or C4)	54	0.87 (0.35–1.46)	44	0.17 (0–0.99)	**0.001**	40
Anti-DNA antibodies	46	1.02 (0.49–1.51)	52	0.16 (0–0.73)	**<0.0001**	24
Fever	6	1.18 (0.76–2.82)	92	0.45 (0.05–1.26)	0.069	0
Thrombocytopenia	3	0.40 (0.18–1.51)	95	0.45 (0.04–1.12)	–	2
Leukopenia	10	0.47 (0.22–1.53)	88	0.44 (0.03–1.12)	0.49	6

C4d levels are presented as median (25–75% quantile), and significance was calculated using the Mann-Whitney *U* test. Significant *p* Values are highlighted in bold. C4d levels were calculated only when more than five patients had the particular symptom at higher disease activity

### C4d levels correlated positively with LN in cross-sectional study

**Fig. 3:**
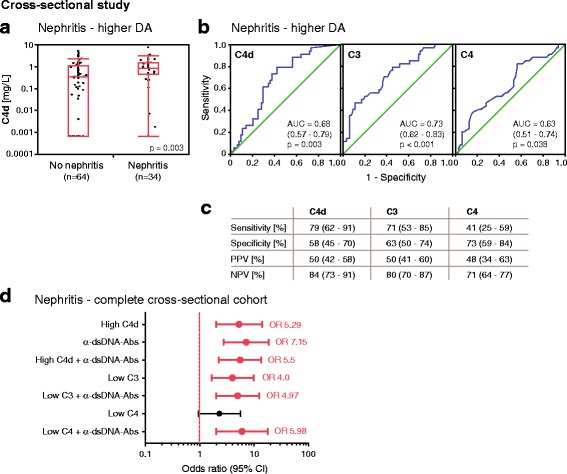
Plasma C4d levels associate with lupus nephritis (LN) in the cross-sectional study and are convincing as an accurate marker for LN. **a** At higher disease activity (DA), plasma C4d levels are elevated in patients with LN (*n* = 34) compared with patients without LN (*n* = 64). Data are presented as medians with 25–75% quantiles plus whiskers, and significance was calculated using the Mann-Whitney *U* test. **b** Area under the ROC curve analysis showing accuracy of C4d, C3, and C4 as markers for LN. **c** Sensitivity, specificity, positive predictive value (PPV), and negative predictive value (NPV) for C4d, C3, and C4 as markers for LN. Data are presented with 95% CIs. **d** Association of high C4d (>0.42 mg/L), low C3 (<0.77 g/L), and low C4 (<0.12 g/L) levels alone as well as in combination with the presence of anti-double-stranded DNA antibodies with LN. Significance was calculated using binary logistics, and ORs are indicated with a dot connected to the 95% CI. Significant ORs are shown in *bold* and marked in *red*

LN is considered a severe manifestation in SLE, and it is strongly associated with complement activation caused by autoantibody deposition in the kidney [9]. In the higher disease activity group, 34 patients had LN. These patients exhibited significantly higher C4d levels than patients without LN (Fig. 3a, Table 2). C3 levels were significantly lower in patients with LN (0.67 g/L, 0.48–0.82 g/L, *p* < 0.001) than in patients without LN (0.91 g/L, 0.71–1.11 g/L). Similar findings were obtained for C4 levels (0.14 g/L, 0.1–0.17 g/L; versus 0.16 g/L, 0.12–0.23 g/L; *p* = 0.038).

The ROC curve analysis revealed that C4d as well as C3 and C4 levels all displayed statistically significant accuracy as markers for LN (Fig. 3b). C4d had the highest sensitivity (79%), PPV (50%), and NPV (84%), whereas C4 exhibited the highest specificity (73%) (Fig. 3c). High C4d levels had a statistically superior accuracy as a marker for nephritis than low C4 levels (*p* = 0.002 by McNemar’s test) and accuracy similar to low C3 levels (*p* = 0.508).

ORs were calculated using generalized estimating equation analysis that corrected for the presence of two samples from each patient to further study these associations for the categorized variables. High C4d levels as well as low C3 levels associated significantly with LN (Fig. 3d). Because the presence of anti-dsDNA antibodies is known to associate with LN [10], we investigated their combined association with high C4d and low C3 levels and determined significant associations (Fig. 3d). Of all other mSLEDAI-qualifying symptoms, C4d levels were only significantly altered in arthritis (Table 2).

### C4d can predict LN in the longitudinal study

**Fig. 4:**
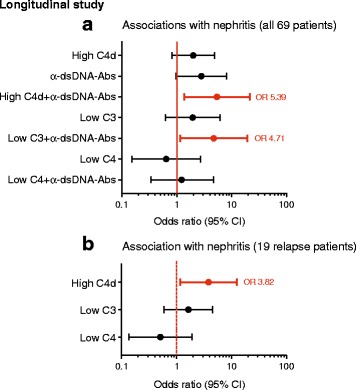
Temporal association of C4d, C3, and C4 with lupus nephritis in the longitudinal study. **a** Temporal association with LN of high C4d (>1.1 mg/L), low C3 (<0.77 g/L), and low C4 (<0.12 g/L) levels alone as well as in combination with the presence of anti-double-stranded DNA (anti-dsDNA) antibodies for all 69 patients within the chosen time interval of 70±30 days. **b** Temporal association with LN for high C4d, low C3, and low C4 levels for the 19 relapse patients within the chosen time interval. Significance was calculated using a generalized estimating equation, and the ORs are indicated with a dot connected to the 95% CI. Significant ORs are shown in *bold* and marked in *red*

An optimal biomarker should predict future flares of specific disease manifestations in order to adjust treatment in time. Thus, using the longitudinal study group, we additionally analyzed whether C4d had the potential to predict future LN within the chosen time interval of 70±30 days. Neither high C4d nor low C3 nor low C4 levels alone could predict LN in the whole cohort. However, together with the presence of anti-dsDNA autoantibodies, high C4d levels showed a significant probability to precede future LN (*p* = 0.016) (Fig. 4a). Low C3 levels in combination with the presence of anti-dsDNA autoantibodies also associated significantly with future LN (*p* = 0.031), whereas no association was found for C4.

Patients with SLE who have once had LN have an increased risk for recurrence of renal flares and generally have a worse prognosis [11]. Therefore, it is important to monitor this patient group vigilantly. During the chosen time interval, 19 of 69 patients had LN on at least one sampling occasion. To study whether C4d could predict future LN in patients with previous occurrence of LN, all available samples collected after at least one occurrence of this manifestation were included (224 samples with 95 renal flares). Only C4d, but neither C3 nor C4, was associated significantly with future LN in the relapse group (*p* = 0.026) (Fig. 4b). High C4d levels alone could not predict any other mSLEDAI-qualifying symptom in the longitudinal study group (data not shown).

## Discussion

In this study, we report that C4d, which is a proteolytic fragment of C4 generated exclusively upon complement activation, appears to be a valuable marker in the follow-up of SLE, in particular in LN. C4d levels correlate with disease activity and rise before renal flares, so that they have the potential to predict recurrence of LN.

Currently used C3 and C4 levels exhibit low sensitivity in follow-up of patients with SLE with broad reference intervals for healthy individuals [12, 13]. C3 and C4 levels are the net result of synthesis and consumption by activation, which are both elevated in inflammation. Accordingly, we observed only a moderate negative correlation of C3 and C4 with C4d, which occurs only as a product of complement activation. Furthermore, C4d levels correlated with mSLEDAI, in which the scores for low complement levels and anti-dsDNA antibodies levels were removed. The absence of C4d in healthy control subjects makes it per se a better marker, and the high fold change increase in higher disease activity, together with the fact that it is more accurate than C4 in discriminating higher disease activity, further supports its suitability. The fact that low complement is listed as a Systemic Lupus Collaborating Clinics classification criterion only to reflect the contribution of complement to SLE pathogenesis, but that it did not improve statistical modeling, further indicates that C3 and C4 are not optimal markers for monitoring SLE [13].

LN, which affects approximately 30–50% of patients with SLE, is one of the most devastating complications in SLE [14]. The pathogenesis of LN involves immune complex deposition [15], which strongly activates complement and gives rise to elevated levels of C4d. It is thus not surprising that C4d is a marker for nephritis in the cross-sectional study, but it is noteworthy that it has a better accuracy than C4 and similar accuracy to C3. Currently, there is still a need for a biomarker that would predict LN so that preventive measures could be initiated as soon as possible. Anti-dsDNA [10] and anti-nucleosome [16] antibodies were suggested as biomarkers for renal disease activity, but they were not convincing as sole biomarkers [17, 18]. C3/C4 levels alone were unable to forecast LN flares [11]. The determination of anti-C1q antibodies is a promising approach, and the measurement of co-occurring anti-C1q and anti-dsDNA antibodies seems most auspicious [19, 20]. In our longitudinal study, only 19 of 69 patients ever had LN during the chosen time interval, which makes it statistically challenging to investigate longitudinal associations. Because anti-dsDNA antibody levels have a certain potential to forecast LN, it was probable that the combination with C4d levels elevated the predictive power of C4d. Patients who have already experienced LN have a high recurrence risk, and it is thus of utmost importance to recognize impending renal flares to avoid organ damage. Especially when considering the low patient numbers, it is striking that the sole measurement of C4d was statistically predictive for recurring LN flares in the relapse group. However, a larger cohort with more patients exhibiting renal disease is needed to confirm and strengthen this observation.

Up to 95% of patients with SLE can have arthritis, which is considered as a mild manifestation [21] not necessarily coinciding with complement activation. Arthritis was associated with low C4d levels, suggesting a different pathogenesis in arthritis in regard to complement activation. This opposing association was seen before in regard to decreased neutrophil extracellular trap degradation [22].

Measurement of C4d, factor Bb, and soluble C5b-9 was previously suggested for the assessment of disease activity and impending flares. Elevated C4d levels showed the highest sensitivity to precede flare [3]. Another study confirmed that C4d and factor Bb are sensitive indicators of moderate to severe lupus disease activity and suggested their measurement especially in patients who, despite evidence of clinical disease activity, do not exhibit altered C3 and C4 levels [4]. One problem with the commercially available C4d ELISA used in both studies is that it is much more prone to detect false-positive readouts arising from sample handling, such as freezing and thawing. Our C4d ELISA has been proven to be much less prone to measure artifacts formed in the course of sample handling and to specifically determine the complement activation-specific cleaved form of C4d [8]. Furthermore, we have shown in a cohort of patients with leukemia treated with anti-CD20 antibodies that levels of C4d detected with our assay clearly correlated with other markers of complement activation, whereas the levels detected with the commercial assay did not [8]. This is due to the recognition of a very short linear neoepitope formed only after C4b cleavage to C4d instead of conformational neoepitopes, which apparently can be mimicked by nonproteolytic events. However, no direct comparison with a commercial assay was done for the patient samples used in the present study.

Another promising approach is the measurement of C4d bound to the surface of various blood cells, such as erythrocytes, reticulocytes, and platelets [23–26]. However, the drawback of this methodology is that it is work-intensive and requires advanced equipment. Because a significant amount of C4d remains soluble in plasma and body fluids [27], we are convinced that measurement by ELISA is more feasible in clinical diagnostics.

## Conclusions

In summary, measuring C4d levels using our robust and practical assay provides a novel method of monitoring SLE activity that is at least as good as C3 and superior to C4 in identifying active disease and in particular LN. Most importantly, C4d levels are more valuable than either C3 or C4 in predicting recurrence of renal flares, aiding clinicians in adjusting treatment in time.

## Abbreviations

ACR: American College of Rheumatology
DA: Disease activity
dsDNA: Double-stranded DNA
ELISA: Enzyme-linked immunosorbent assay
LN: Lupus nephritis
mSLEDAI: Modified Systemic Lupus Erythematosus Disease Activity Index
NPV: Negative predictive value
PPV: Positive predictive value
SLE: Systemic lupus erythematosus
SLEDAI: Systemic Lupus Erythematosus Disease Activity Index
SLEDAI-2K: Systemic Lupus Erythematosus Disease Activity Index 2000
